# Supplementary material for: Benchmarking evolutionary tinkering underlying human–viral molecular mimicry shows multiple host pulmonary–arterial peptides mimicked by SARS-CoV-2
Source: Cell Death Discov. 2020 Oct 2;6:96. doi: 10.1038/s41420-020-00321-y (PMC7529588; doi:10.1038/s41420-020-00321-y)
Supplement: Supplementary file 1 — Supplementary Figure and Table Legends [file 41420_2020_321_MOESM1_ESM.pdf]

Supplementary Legends

Supplementary Table Legends

Table S1. Reference SARS-CoV-2 proteome from UniProt

Table S2: All 33 peptides that are shared between SARS-CoV-2 and the human proteome

Table S3. Distinctive peptides from SARS-CoV-2, not present in previously sequenced human coronavirus strains, that do mimic human proteins. There is no compelling positive T-cell immune response against either the human or viral proteins to warrant further discussion in the current study, but these will be the topic of follow-up experimental studies into SARS-CoV-2-based immunologic modulation in humans.

Table S4. Reference SARS-CoV proteome from UniProt

Table S5. Seasonal human coronavirus (HCoV) peptide mimicry of human proteins with experimental evidence of positive T-cell assays with specific MHC restriction. The MHC-TCR-peptide assays conducted include: (Assay 2.1) Cellular MHC/mass spectrometry, ligand presentation (Assay 2.2)

Table S6. SARS-CoV peptide mimicry of human proteins with experimental evidence of positive T-cell assays with specific MHC restriction. The MHC-TCR-peptide assays conducted include: (Assay 2.1) Cellular MHC/mass spectrometry, ligand presentation (Assay 2.2)

Table S7. MERS peptide mimicry of human proteins with experimental evidence of positive T-cell assays with specific MHC restriction. The MHC-TCR-peptide assays conducted include: (Assay 2.1) Cellular MHC/mass spectrometry, ligand presentation (Assay 2.2)

Supplementary Figure Legends

Figure 1 – Supplementary Figure 1. Mimicked 8-mers/9-mers between human and viral proteomes (a) Distribution of mimicked 8-mers/9-mers between human and viral proteomes. (b) Distribution of mimicked 8-mers/9-mers between human and viral proteomes normalized by the number of unique 8-mers/9-mers.

Figure 3 – Supplementary Figure 1. Single cell RNA-seq results for ANXA7

Figure 3 – Supplementary Figure 2. Human ANXA7 mimicking peptide ESGLKTIL is only present in SARS-CoV-2, with the closest known evolutionary homologs being from BAT SARS-like coronavirus (ESGLKTIL), pangolin coronavirus, and the NL63-related bat coronavirus strains.
