# Supplementary material for: Benchmarking evolutionary tinkering underlying human–viral molecular mimicry shows multiple host pulmonary–arterial peptides mimicked by SARS-CoV-2
Source: Cell Death Discov. 2020 Oct 2;6:96. doi: 10.1038/s41420-020-00321-y (PMC7529588; doi:10.1038/s41420-020-00321-y)
Supplement: Supplementary file 2 — Supplementary Material [file 41420_2020_321_MOESM2_ESM.pdf]

Supplementary Material

Table S1. Reference SARS-CoV-2 proteome from UniProt

SARS-CoV-2 Protein Name	SARS-CoV-2 Gene Symbol	UniProt ID (Length)
R1AB_SARS2 Replicase polyprotein 1ab	rep	P0DTD1 (7096)
SPIKE_SARS2 Spike glycoprotein	S	P0DTC2 (1273)
R1A_SARS2 Replicase polyprotein	rep	P0DTC1 (4405)
NS7A_SARS2 Protein 7a	7a	P0DTC7 (121)
AP3A_SARS2 Protein 3a	3a	P0DTC3 (275)
VME1_SARS2 Membrane protein	3	P0DTC5 (222)
NCAP_SARS2 Nucleoprotein	N	P0DTC9 (419)
ORF9B_SARS2 Protein 9b	3	P0DTD2 (97)
VEMP_SARS2 Envelope small membrane protein	E	P0DTC4 (75)
NS6_SARS2 Non-structural protein 6	6	P0DTC6 (61)
NS8_SARS2 Non-structural protein 8	8	P0DTC8 (121)
NS7B_SARS2 Protein non-structural 7b	7b	P0DTD8 (43)
Y14_SARS2 Uncharacterized protein 14	ORF14	P0DTD3 (73)
A0A663DJA2_SARS2 ORF10	ORF10	A0A663DJA2 (38)

Table S2: All 33 peptides that are shared between SARS-CoV-2 and the human proteome

Peptide	Viral Protein	Accession No.	Start	End	Human Protein	Description	Uniprot ID	Start	End
AKKNNLPF	R1AB_SARS2 Replicase polyprotein 1ab	P0DTD1	2732	2739	LPGAT1	Lysophosphatidylglycerol Acyltransferase 1	Q92604	198	205
DEDDSEPV	SPIKE_SARS2	P0DTC2	1256	1263	MYO16	Myosin XVI	Q9Y6X6	1403	1410
DEDEEEGD	R1AB_SARS2 Replicase polyprotein 1ab	PODTD1	927	934	GMCL1	Germ Cell-Less 1, Spermatogenesis Associated	Q96IK5	68	75
DIQLLKSA	R1AB_SARS2 Replicase polyprotein 1ab	P0DTD1	1126	1133	EML1	EMAP Like 1	O00423	50	57
DTSLSGFK	R1AB_SARS2 Replicase polyprotein 1ab	P0DTD1	3670	3677	SLC12A7	Solute Carrier Family 12 Member 7	Q9Y666	994	1001
ELPDEFVV	ORF9B_SARS2 Protein 9b	P0DTD2	85	92	MROH2B	Maestro Heat Like Repeat Family Member 2B	Q7Z745	102	109
ESGLKTIL	R1AB_SARS2 Replicase polyprotein 1ab	P0DTD1	389	396	ANXA7	Annexin A7	P20073	403	410
EVEKGVLP	R1AB_SARS2 Replicase polyprotein 1ab	P0DTD1	54	61	NDST1	N-Deacetylase And N-Sulfotransferase 1	P52848	213	220
GPPGTGKS	R1AB_SARS2 Replicase polyprotein 1ab	P0DTD1	5605	5612	SETX	Senataxin	Q7Z333	1962	1969
					VPS4A	Vacuolar Protein Sorting 4 Homolog A	Q9UN37	166	173
					VPS4B	Vacuolar Protein Sorting 4 Homolog B	O75351	173	180
KDKKKKAD	NCAP_SARS2 Nucleoprotein	P0DTC9	369	376	MICAL3	Microtubule Associated Monooxygenase, Calponin And LIM Domain Containing 3	Q7RTP6	1748	1755
KKDKKKKA	NCAP_SARS2 Nucleoprotein	P0DTC9	368	375	MICAL3	Microtubule Associated Monooxygenase, Calponin And LIM Domain Containing 3	Q7RTP6	1747	1754
KKDKKKKAD	NCAP_SARS2 Nucleoprotein	P0DTC9	368	376	MICAL3	Microtubule Associated Monooxygenase, Calponin And LIM Domain Containing 3	Q7RTP6	1747	1755
LALITLAT	NS7A_SARS2 Protein 7a	P0DTC7	6	13	HTR1B	5-Hydroxytryptamine Receptor 1B	P28222	55	62
LVDPQIQL	ORF9B_SARS2 Protein 9b	P0DTD2	13	20	VARS2	Valyl-TRNA Synthetase 2, Mitochondrial	Q5ST30	988	995
NVAITRAK	R1AB_SARS2 Replicase polyprotein 1ab	PODTD1	5885	5892	DNA2	DNA Replication Helicase/Nuclease 2	P51530	1000	1007
PDEDEEEG	R1AB_SARS2 Replicase polyprotein 1ab	P0DTD1	926	933	CC2D1A	Coiled-Coil And C2 Domain Containing 1A	Q6P1N0	83	90
PGSGVPVV	R1AB_SARS2 Replicase polyprotein 1ab	P0DTD1	4618	4625	PAM	Peptidylglycine Alpha-Amidating Monooxygenase	P19021	859	866
QGPPGTGK	R1AB_SARS2 Replicase polyprotein 1ab	P0DTD1	5604	5611	HELZ2	Helicase With Zinc Finger 2	Q9BYK8	2172	2179
					UPF1	UPF1 RNA Helicase And ATPase	Q92900	501	508

					ZNFX1	Zinc Finger NFX1-Type Containing 1	Q9P2E3	617	624
RFNVAITR	R1AB_SARS2 Replicase polyprotein 1ab	P0DTD1	5883	5890	MOV10L1	Mov10 Like RISC Complex RNA Helicase 1	Q9BXT6	1130	1137
RRARSVAS	SPIKE_SARS2 Spike glycoprotein	P0DTC2	681	688	SCNN1A	Sodium Channel Epithelial 1 Subunit Alpha	P37088	200	207
RRSFYVYA	R1AB_SARS2 Replicase polyprotein 1ab	P0DTD1	2430	2437	TPRA1	Transmembrane Protein Adipocyte Associated 1	Q86W33	224	231
RYPANSIV	R1AB_SARS2 Replicase polyprotein 1ab	P0DTD1	6315	6322	BRI3	Brain Protein I3	O95415	65	72
SLKELLQN	R1AB_SARS2 Replicase polyprotein 1ab	P0DTD1	3529	3536	CENPI	Centromere Protein I	Q92674	495	502
SRSSSRSR	NCAP_SARS2 Nucleoprotein	P0DTC9	183	190	CCNL2	Cyclin L2	Q96S94	462	469
					CLASRP	CLK4 Associating Serine/Arginine Rich Protein	Q8N2M8	395	402
					LUC7L2	LUC7 Like 2, Pre-MRNA Splicing Factor	Q9Y383	304	3011
SSRSSSRS	NCAP_SARS2 Nucleoprotein	P0DTC9	182	189	CLASRP	CLK4 Associating Serine/Arginine Rich Protein	Q8N2M8	390	397
					LUC7L2	LUC7 Like 2, Pre-MRNA Splicing Factor	Q9Y383	304	311
SSRSSSRS R	NCAP_SARS2 Nucleoprotein	P0DTC9	182	190	CLASRP	CLK4 Associating Serine/Arginine Rich Protein	Q8N2M8	394	402
					PGD	Phosphogluconate Dehydrogenase	P52209	277	284
VNSVLLFL	VEMP_SARS2 Envelope small membrane protein	P0DTC4	13	20	RANBP6	RAN Binding Protein 6	O60518	408	415
VTLIGEAV	R1AB_SARS2 Replicase polyprotein 1ab	P0DTD1	6616	6623	PGD	Phosphogluconate Dehydrogenase	P52209	277	284
YNYEPLTQ	R1AB_SARS2 Replicase polyprotein 1ab	P0DTD1	3499	3506	MCM8	Minichromosome Maintenance 8 Homologous Recombination Repair Factor	Q9UJA3	198	205
EVLLAPLL	R1AB_SARS2 Replicase polyprotein 1ab	P0DTD1	1141	1148	ARL6IP4	ADP Ribosylation Factor Like GTPase 6 Interacting Protein 4	ENSP00 0004389 69.1	175	182
PEANMDQE	R1AB_SARS2 Replicase polyprotein 1ab	P0DTD1	4311	4318	ALOX5AP	Arachidonate 5-Lipoxygenase Activating Protein	ENSP00 0004798 70.1	53	60
GGSCVLSG	R1AB_SARS2 Replicase polyprotein 1ab	P0DTD1	1099	1106	SNX27	Sorting Nexin 27	ENSP00 0004967 75.1	111	118
REETGLLM	R1AB_SARS2 Replicase polyprotein 1ab	P0DTD1	723	730	ESRRG	Estrogen Related Receptor Gamma	ENSP00 0004663 43.1	723	730

Table S3. Distinctive peptides from SARS-CoV-2, not present in previously sequenced human coronavirus strains, that do mimic human proteins. There is no compelling positive T-cell immune response against either the human or viral proteins to warrant further discussion in the current study, but these will be the topic of follow-up experimental studies into SARS-CoV-2-based immunologic modulation in humans.

SARS-CoV-2 Peptide	SARS-CoV-2 protein	Mimicked Human Protein
AKKNNLPF (SARS-CoV-2)	NSP3 (YP_009725299.1 : 1914-1921; P0DTD1:2732-2739)	**LPGAT1** (Q92604:198-205)
DEDEEEGD (SARS-CoV-2)	NSP3 (YP_009725299.1 : 109-116; P0DTD1: 927-934)	**GMCL1** (Q96IK5: 68-75)
DIQLLKSA (SARS-CoV-2)	NSP3 (YP_009725299.1 : 50-57; P0DTD1:1126-1133)	**EML1** (O00423: 50-57)
DTSLSGFK (SARS-CoV-2)	NSP6 (YP_009725302.1 :101-108; P0DTD1: 3670-3677)	**SLC12A7** (Q9Y666:994-1001)
EVEKGVLP (SARS-CoV-2)	Leader protein (YP_009725297.1 :54-61; P0DTD1:54-61)	**NDST1** (P52848:213-220)
KKDKKKKAD (SARS-CoV-2)	Nucleocapsid phosphoprotein (YP_009724397.2 : 368-37; P0DTC9:368-376)	**MICAL3** (Q7RTP6: 368-376**)**
LALITLAT (SARS-CoV-2)	ORF7a protein (YP_009724395.1 : 6-13; P0DTC7:6-13)	**HTR1B** (P28222: 55-62)
PDEDEEEG (SARS-CoV-2)	NSP3 (YP_009725299.1 :108-115; P0DTD1:926-933)	**CC2D1A** (Q6P1N0:83-90)
RRARSVAS (SARS-CoV-2)	Surface glycoprotein (YP_009724390.1 :681-688; P0DTC2:681-688)	**SCNN1A** (P37088: 200-207)
RRSFYVYA (SARS-CoV-2)	NSP3 (YP_009725299.1 :1612-1619; P0DTD1:2430-2437)	**TPRA1** (Q86W33:224-231)
RYPANSIV (SARS-CoV-2)	3'-to-5' exonuclease (YP_009725309.1 : 390-397; P0DTD1:6315-6322)	**BRI3** (O95415: 65-72)

Table S4. Reference SARS-CoV proteome from UniProt

SARS-CoV Protein Name	SARS-CoV Gene Symbol	UniProt ID
R1AB_CVHSA<br>Replicase polyprotein 1ab	rep	P0C6X7<br>(7073)
SPIKE_CVHSA<br>Spike glycoprotein	S	P59594<br>(1255)
R1A_CVHSA<br>Replicase polyprotein	rep	P0C6U8<br>(4382)
NS7A_CVHSA<br>Protein 7a	7a	P59635<br>(122)
AP3A_CVHSA<br>Protein 3a	3a	P59632<br>(274)
VME1_CVHSA<br>Membrane protein	3	P59596<br>(221)
NCAP_CVHSA<br>Nucleoprotein	N	P59595<br>(422)
NS3B_CVHSA<br>Non-structural protein 3b	3b	P59633<br>(154)
ORF9B_CVHSA<br>Protein 9b	3	P59636<br>(98)
VEMP_CVHSA<br>Envelope small membrane protein	E	P59637<br>(76)
NS6_CVHSA<br>Non-structural protein 6	6	P59634<br>(63)
NS8B_CVHSA<br>Non-structural protein 8b	8b	Q80H93<br>(84)
NS8A_CVHSA<br>Non-structural protein 8	8a	Q7TFA0<br>(39)
NS7B_CVHSA<br>Protein non-structural 7b	7b	Q7TFA1<br>(44)
Y14_CVHSA<br>Uncharacterized protein 14	ORF14	Q7TLC7<br>(70)

Table S5. Seasonal human coronavirus (HCoV) peptide mimicry of human proteins with experimental evidence of positive T-cell assays with specific MHC restriction. The MHC-TCR-peptide assays conducted include: (Assay 2.1) Cellular MHC/mass spectrometry, ligand presentation {ref}, (Assay 2.2)

Viral Peptide	Viral Protein (HCoV strain: Uniprot)	Human Epitope	Human Protein	MHC restriction (Assay - T-cell stimulation)	Epitope ID (IEDB)	Pubmed ID (PMID)
GPPGTGKS (HKU1;OC43)	Nsp13 (**HCoV-HKU1** - YP_459942.1 :280-287) ; Nsp10 (**HCoV-OC43** - YP_009555254.1:280-287)	GPPGTGKSYLAKAVATEAN	SKD1 (167-185)	HLA-DRA*01:01 HLA-DRB1*08:01 **(Assay 2.1 - Positive)**	433968	21654843
GRIVTLIS (HKU1)	Nsp6 (YP_460019.1: 142-149)	GRIVTLISF	MCL-1 (262-270)	HLA-B*27:05 HLA-B*27:09 HLA-B*27:04 HLA-B*27:01 HLA-B*27:02 HLA-B*27:06 HLA-B*27:07 HLA-B*27:08 **(Assay 2.1 - Positive)**	241225	31844290 31154438 29632046 29393594 28188227 28063628 27920218 26811146 27846572 26992070 26929215 25469497 26154972 25418920 25645385 20112406
SLLRTSIM (NL63)	Replicase polyprotine 1ab (HCoV-NL63 YP_003766.2 :1920-1927)	SLLRTSIMSK	CCT8 (162-171)	HLA class I cellular MHC/mass spectrometry ligand presentation **Positive**	625570	26992070
TCNSKLTL (OC43)	Spike glycoprotein (YP_009555241.1: 254-261)	STCNSKLTLK	LIM (H0Y592:112-121)	HLA class I mass spectrometry ligand presentation **Positive**	884003	30429286
VVGSTEEVK (229E)	Replicase polyprotine 1ab (NP_073549.1: 524-532)	HLPFAVVGSTEEVKIGNK	SEPTIN11 (Q9NVA2:241-258)	HLA-B*27:05	799404	29393594

Table S6. SARS-CoV peptide mimicry of human proteins with experimental evidence of positive T-cell assays with specific MHC restriction. The MHC-TCR-peptide assays conducted include: (Assay 2.1) Cellular MHC/mass spectrometry, ligand presentation {ref}, (Assay 2.2)

Viral Peptide	Viral Protein (SARS-CoV: Uniprot)	Human Epitope	Human Protein	MHC restriction (Assay - T-cell stimulation)	Epitope ID (IEDB)	Pubmed ID (PMID)
GPPGTGKS (SARS-CoV; MERS; HCoV-OC43; HCoV-HKU1)	Nsp13 (NP_828870.1 :281-288)	GPPGTGKSYLAKAVATEAN	**VPS4A** (Q9UN37 :167-185)	HLA-DRA*01:01 HLA-DRB1*08:01 **(Assay 2.1 - Positive)**	433968	21654843
YNYEPLTQ (SARS-CoV; SARS-CoV-2)	Nsp5 (NP_828863.1 :236-243)	RVYNYEPLTQLK	**MCM8** (Q9UJA3: 197-208)	HLA-A*03:01 cellular MHC/mass spectrometry ligand presentation **Positive**	624802	31844290 30315122 28228285 26992070

Table S7. MERS peptide mimicry of human proteins with experimental evidence of positive T-cell assays with specific MHC restriction. The MHC-TCR-peptide assays conducted include: (Assay 2.1) Cellular MHC/mass spectrometry, ligand presentation {ref}, (Assay 2.2)

Viral Peptide	Viral Protein (MERS-CoV: Uniprot)	Human Epitope	Human Protein	MHC restriction (Assay - T-cell stimulation)	Epitope ID (IEDB)	Pubmed ID (PMID)
DGKPISAY (MERS-CoV)	Nsp2 (YP_009047214.1 :12-19)	LENFYPLEGGRVLLDGKPISAYD	ABCB9 (Q9NP78:552-574)	HLA-A*30:02 (mass spectrometry ligand presentation **Positive**)	1028898	31844290
GPPGTGKS (SARS-CoV-2; SARS-CoV; MERS-CoV; HCoV-OC43; HCoV-HKU1)	Nsp13 (YP_009047224.1: 281-288)	GPPGTGKSYLAKAVATEAN	VPS4A (Q9UN37:167-185)	HLA-DRA*01:01 HLA-DRB1*08:01 **(Assay 2.1 - Positive)**	433968	21654843
LLGSIAGV	Spike glycoprotein (YP_009047204.1: 950:957)	GLLGSIAGV	CLCF1 (Q9UBD9: 142-150)	HLA-Class I cellular MHC/mass spectrometry ligand presentation **Positive**	923359	31222486<br>31154438
