# Supplementary figures and images for: Benchmarking evolutionary tinkering underlying human–viral molecular mimicry shows multiple host pulmonary–arterial peptides mimicked by SARS-CoV-2
Source: Cell Death Discov. 2020 Oct 2;6:96. doi: 10.1038/s41420-020-00321-y (PMC7529588; doi:10.1038/s41420-020-00321-y)

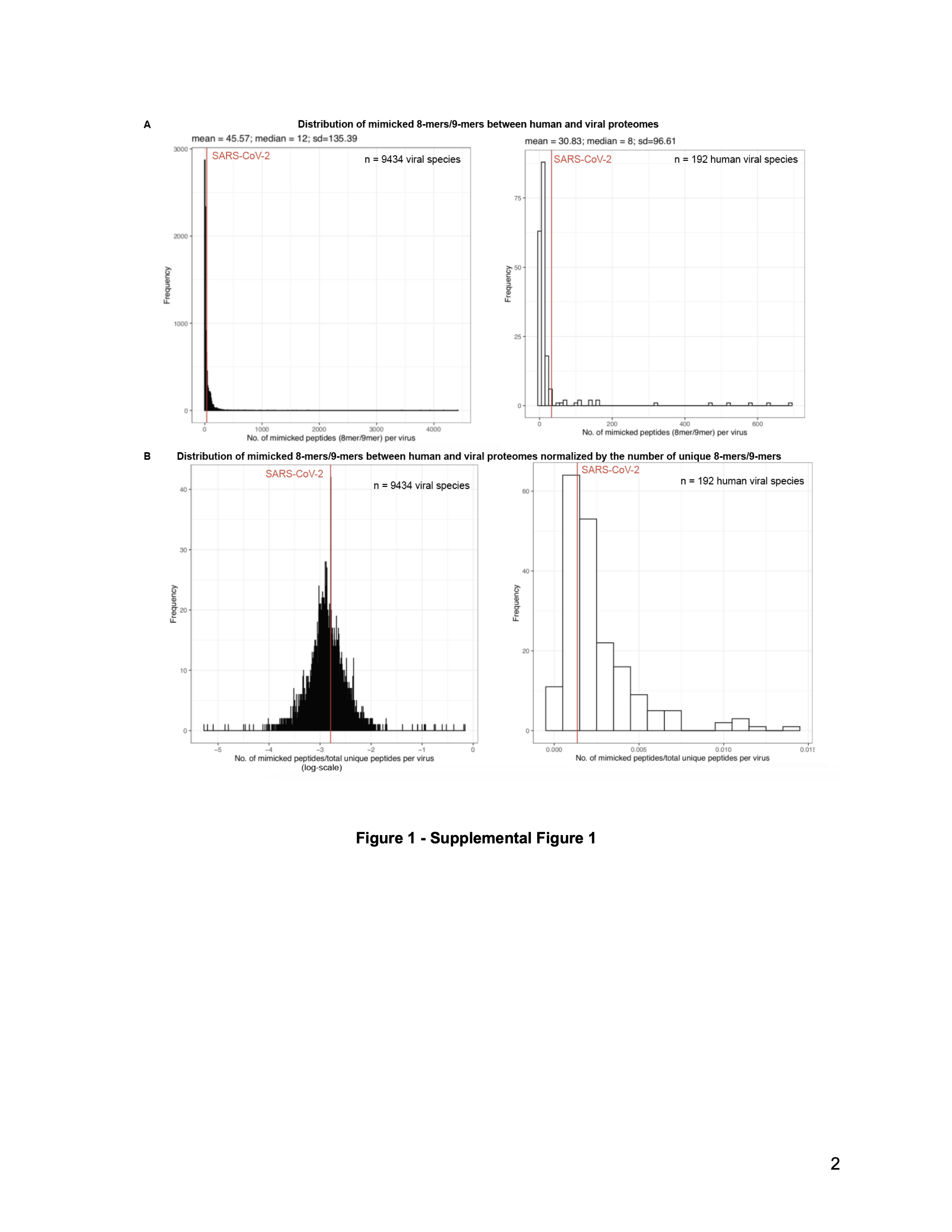

Supplement: Supplementary file 3 — Supplementary Figure [file 41420_2020_321_MOESM3_ESM.png]

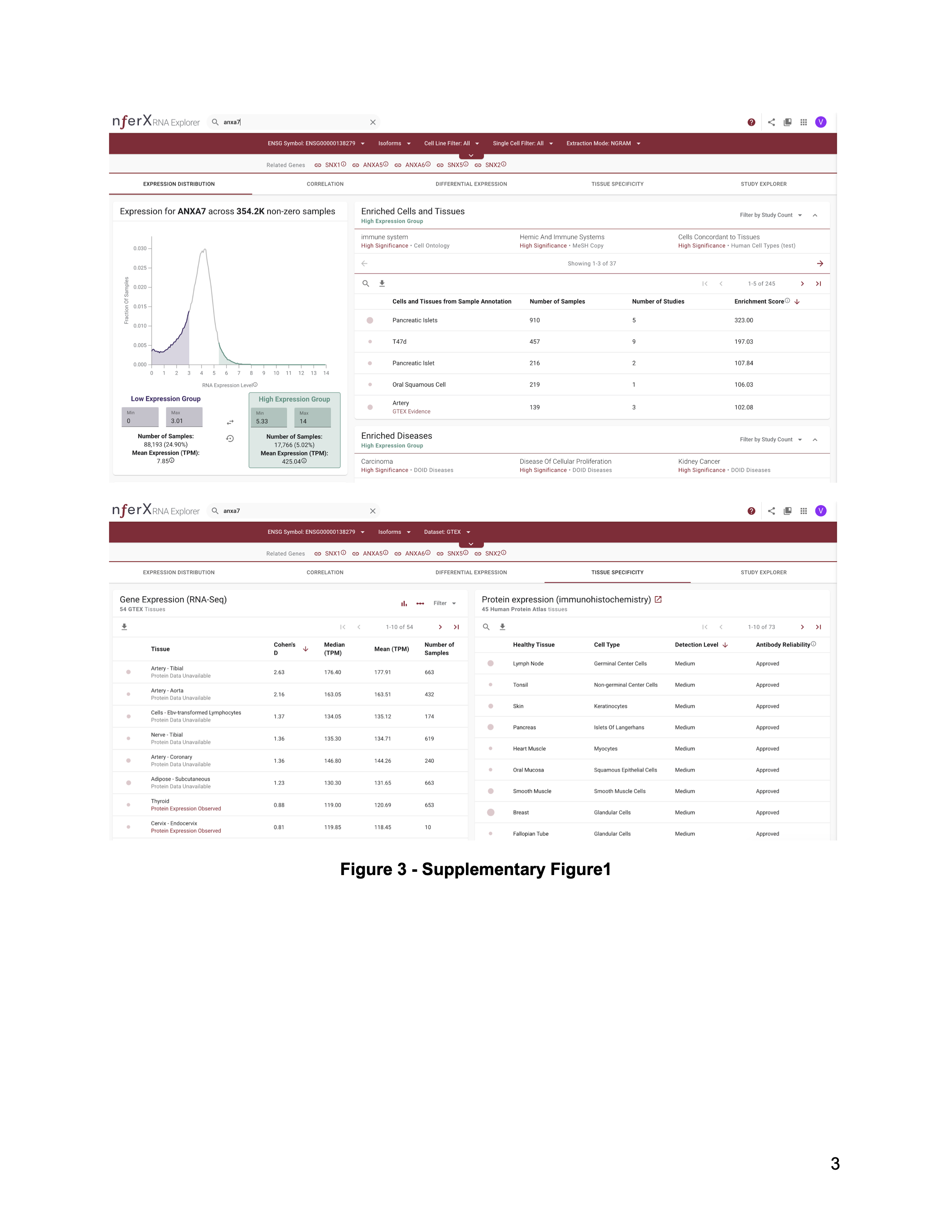

Supplement: Supplementary file 4 — Supplementary Figure [file 41420_2020_321_MOESM4_ESM.png]

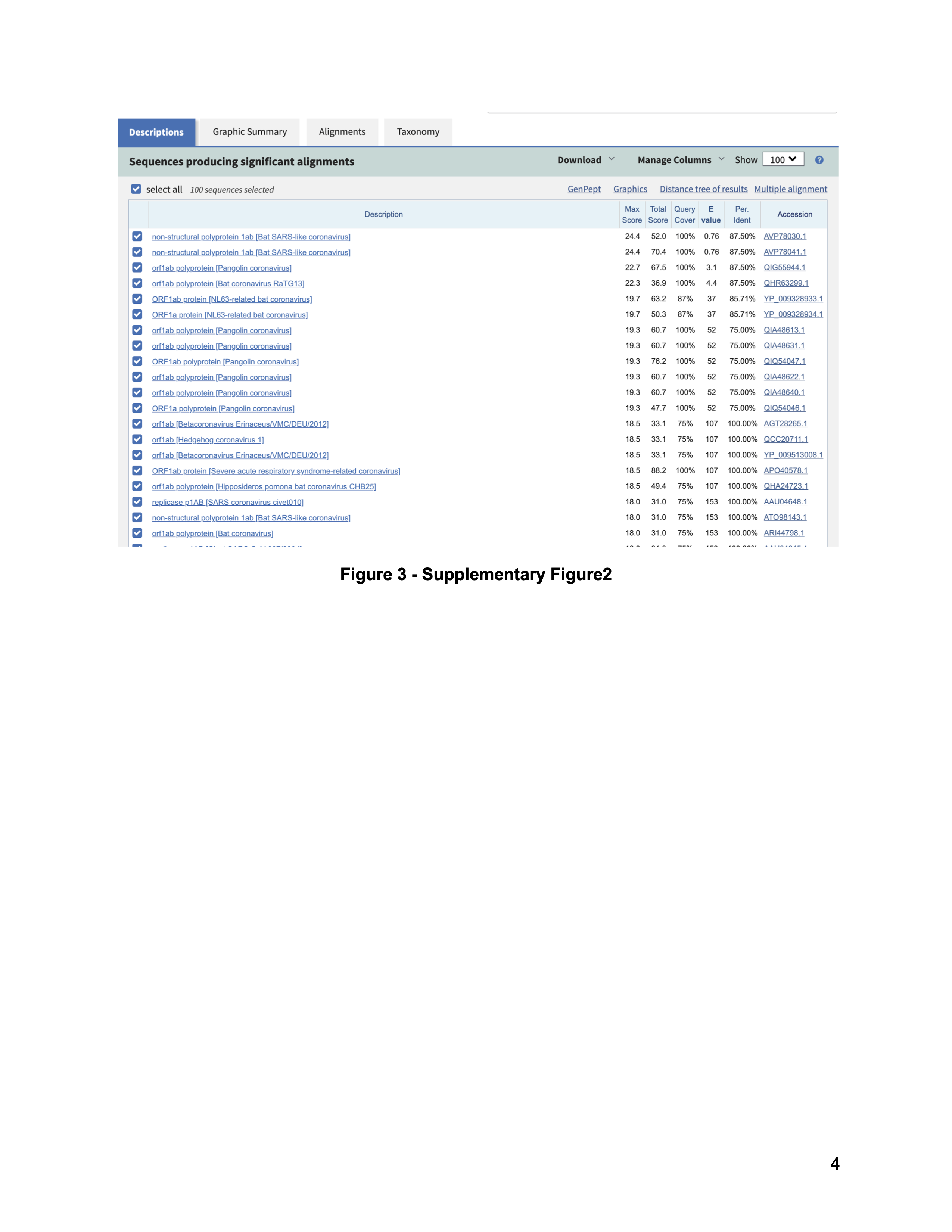

Supplement: Supplementary file 5 — Supplementary Figure [file 41420_2020_321_MOESM5_ESM.png]
